# Skeletal muscle analysis of panoramic ultrasound is reliable across multiple raters

**DOI:** 10.1371/journal.pone.0267641

**Published:** 2022-05-02

**Authors:** Christopher J. Cleary, Omid Nabavizadeh, Kaycie L. Young, Ashley A. Herda

**Affiliations:** 1 Department of Health, Sport, and Exercise Sciences, University of Kansas Edwards Campus, Overland Park, Kansas, United States of America; 2 Department of Geriatric Medicine, University of Colorado-Anschutz Medical Campus, Aurora, Colorado, United States of America; University of Mississippi, UNITED STATES

## Abstract

Ultrasound devices are common in muscle physiology laboratories due to their ease of use and validity to assess skeletal muscle characteristics. The current study assessed the reliability of ultrasound skeletal muscle image analysis across multiple raters with limited experience. Vastus lateralis (VL), rectus femoris (RF), and first dorsal interosseus (FDI) images were separately analyzed by three novice raters to determine muscle thickness (MT), cross-sectional area (CSA), and echo-intensity (EI). Separate analyses of variance (ANOVA) assessed statistical differences between and within raters. Intra-class correlation coefficients (ICC) between (inter-rater) and within (intra-rater) raters, the standard error of the measurement (SEM) and minimal difference needed to be considered real were calculated. Inter-rater reliability was high for the VL and RF (ICC: 0.984–0.999), while the FDI was lower (0.614–0.962). Further, intra-rater reliability was greater than 0.961 for each rater. SEM values calculated for inter-rater reliability expressed as a percentage of the mean ranged from 0.4–5.8% across variables. Similarly, SEM values for intra-rater reliability were between 0.8–5.8%, 0.6–3.6%, and 0.4–3.2% for Raters 1, 2 and 3, respectively. Despite this, significant differences (*p*<0.05) between raters were observed for RF MT and EI, VL CSA and EI, and FDI MT, suggesting that potentially more measurement trials or greater practice time may be necessary to reduce systematic error among multiple raters. Post-image acquisition processing is reliable among and within raters as determined through ICCs and SEMs. This study provided consistent results among three separate novice raters given the same training, a unique yet realistic setting in muscle physiology laboratories.

## Introduction

Ultrasound imaging is a non-invasive and widely available modality to assess skeletal muscle characteristics such as muscle thickness (MT), muscle cross-sectional area (CSA), and echo intensity (EI) [1, 2]. Ultrasound devices are less cumbersome, inexpensive, portable, and potentially safer than computed topography (CT) or magnetic resonance imaging (MRI) such that that there is no radiation exposure associated with ultrasound when used for similar musculoskeletal applications. For skeletal muscle assessments, ultrasound imaging can evaluate the efficacy of a rehabilitation program following surgery [3], monitor rates of atrophy during bed rest [4], or assess adaptations derived from resistance training programs [5, 6]. Traditional ultrasound assessment methods, such as use of longitudinal view, have a smaller field of view-only as wide as the probe dimensions, which could potentially limit the application to multiple muscles of different sizes and the ability to measure full skeletal muscle CSA in muscles that span more than 4 cm. However, advanced scanning technology with extended fields of view, deemed panoramic ultrasound imaging, has been recently utilized to allow muscles with larger CSAs to be examined [7, 8]. Panoramic ultrasound has also been previously validated against other skeletal muscle imaging modalities, such as MRI and CT [9]. Ahtiainen et al. [5] examined the vastus lateralis (VL) muscle CSA via MRI and panoramic ultrasound before and after a 21-week lower-body resistance training intervention and reported comparable values across imaging devices. When compared to CT, panoramic ultrasound of the quadriceps muscle CSA had minimal (1.3–2.5%) differences between techniques, with excellent intra-class correlation coefficients (ICC) of 0.951 to 0.998 [10]. Use of ultrasound can occur in a clinical or laboratory setting without radiation or magnet restrictions, and, aside from the initial purchase, operation is inexpensive to the user. As such, the high level of agreement among these differing methods may allow smaller-scaled laboratories to cost-effectively quantify muscle (CSA, MT, EI, etc.) in research settings when there is no access to MRI or CT devices [11].

As Hernández-Belmonte et al. and others point out, during ultrasound assessment for skeletal muscle characteristics, the experience and skill level of both sonographer and the analyst/rater (those analyzing the image offline) can influence the reliability and validity of the quantitative variables recorded [12–14]. For the sonographer, factors such as probe orientation and skin compression are some, but not all, of the factors influencing accuracy of image acquisition [8, 13, 15]. In a research setting, consistency among sonographers is critical and typically performed by an experienced senior individual having imaged an innumerable manifest of individuals of all different shapes and sizes. However, equally important is the post-image processing and particularly the individuals analyzing the acquired images [15], where human anatomy, knowledge of ultrasound devices, and previous experience with image analysis software can influence accuracy [16]. Due to this, previous studies have assessed the difference between multiple raters (inter-rater reliability) [1, 2] and within a single rater (intra-rater reliability) [16] for assessing skeletal muscle ultrasound images, often only with two raters. For better application to research settings, and accurately quantify ultrasound-derived variables’ mean values such as CSA or MT [17] and to adequately report image analysis reliability [18], greater than two raters may be needed. However, more than two raters have not been investigated in the current literature.

Further, the reliability of panoramic ultrasound for various skeletal muscles of the lower and upper body have not been investigated collectively. For example, the first dorsal interosseus (FDI) has been assessed to investigate neuromuscular function in diseased and healthy populations [19, 20], yet no studies have assessed the reliability of FDI panoramic ultrasound, nor concurrently compare the ICCs of upper and lower limb muscles. The quadriceps muscles are much larger and generally have well-defined borders or aponeuroses, whereas some thoracic and upper limb (forearm) muscles [15] are not as easy to differentiate from neighboring tissue due to their size and anatomical arrangement [18]. These differences emphasize the importance of experienced sonographers for image acquisition and analysts that are familiar with the anatomical properties of separate muscles.

As the importance of the skill of the sonographer is now established [14], as well as the individual(s) analyzing the images, we seek to identify the consistency of the rater. As laboratory groups may bring in new students or laboratory assistants over time and to increase the accuracy of these novice raters, standardized instructions should be utilized. Therefore, using specific, written, and established guidance on how to analyze a panoramic ultrasound image that are provided to novice raters, the purpose of this study was to evaluate the intra- and inter-rater reliability of ultrasound image analysis from the VL, RF, and FDI using a pre-determined set of written guidelines among multiple raters using images captured by skilled and experienced sonographers.

## Materials and methods

This study included image analysis from three separate IRB-approved protocols including healthy, active, college-aged (18–35-year-old) males and females without any presentation of musculoskeletal disorders. Data were acquired in an observational design with the purpose of analyzing previously collected, deidentified ultrasound images of the VL, RF, and FDI. Panoramic ultrasound images of the VL (n = 15), RF (n = 15), and the FDI (n = 12) were assessed by each of three raters who analyzed the images in triplicate for MT, CSA, and EI. Participant characteristics could not be summarized as the images analyzed were previously deidentified for the purposes of this study. All images were previously acquired from participants that signed written statements of informed consent from unrelated studies, and the retrospective procedures were approved by the University Human Research Protection Program/IRB (#STUDY000146569) in accordance with the Declaration of Helsinki. All experiments were performed in accordance with relevant guidelines and regulations.

### Procedures

#### Image acquisition

Specific replicable details on image acquisition can be found in each study’s respective publications (*references blinded for review*). As stated above, descriptive statistics were not summarized for this study, as image analysis was the focus of this project. Further, all images had been completely deidentified from any subject ID codes. Acquisition methods were identical and conducted within the same laboratory group of researchers. The RF and VL images were captured by one senior investigator with over 5-years of musculoskeletal ultrasound experience (>1000 examinations) and the FDI images were captured by a colleague who was identically trained with 4-years of musculoskeletal ultrasound experience (>1000 examinations).

Briefly, the VL and RF images were captured using a LOGIQ e ultrasound device (GE Healthcare, Wauwatosa, WI, USA) with a linear array transducer (Model L4-12t-RS, 4.2–30 MHz). Images were acquired on the right leg for all participants while the subjects laid supine on an examination table. The participants rested in a supine position for 10 minutes prior to image acquisition to allow for the shifting of fluids and the leg was slightly elevated by placing cushions under the calf to allow slight knee and hip flexion and allow probe access to posteriolateral aspects of the VL. Ultrasound scan depth was 6 cm, gain was 68 dB, and transducer frequency was 10 MHz to optimize image quality for all participants. For these studies, the RF images were captured at 50% of the distance between the anterior superior iliac spine and the superior pole of the patella [21] while VL images were captured at 50% of the length between the greater trochanter of the femur and lateral epicondyle of the femur [5].

The FDI images were captured using a separate LOGIQ e ultrasound device (GE Healthcare, Wauwatosa, WI, USA) with a linear array transducer (Model 12L-RS, 5–13 MHz). Participants were seated during FDI measurements, and the right hand was placed on the examination table on top of foam pads to elevate the hand from the table. Scan depth was 4 cm, gain was 38 dB and transducer frequency was 12 MHz. All images were captured on the right hand at the midpoint of the origin and insertion of the FDI. The location of the FDI was identified through a single longitudinal scan, but the image acquisition occurred in the panoramic mode as not all participant’s FDIs fit within the single probe width [22]. For all panoramic scans, an ample amount of water-soluble transmission gel was applied to the skin and the transducer to enhance image quality and provide optimal acoustic coupling. The transducer was moved manually across the selected muscle in the transverse plane with a constant, minimal pressure to acquire a full view of the skeletal muscles and consistent capture. All images were saved to an external storage device and exported for subsequent analyses.

#### Image analysis

**Fig 1:**
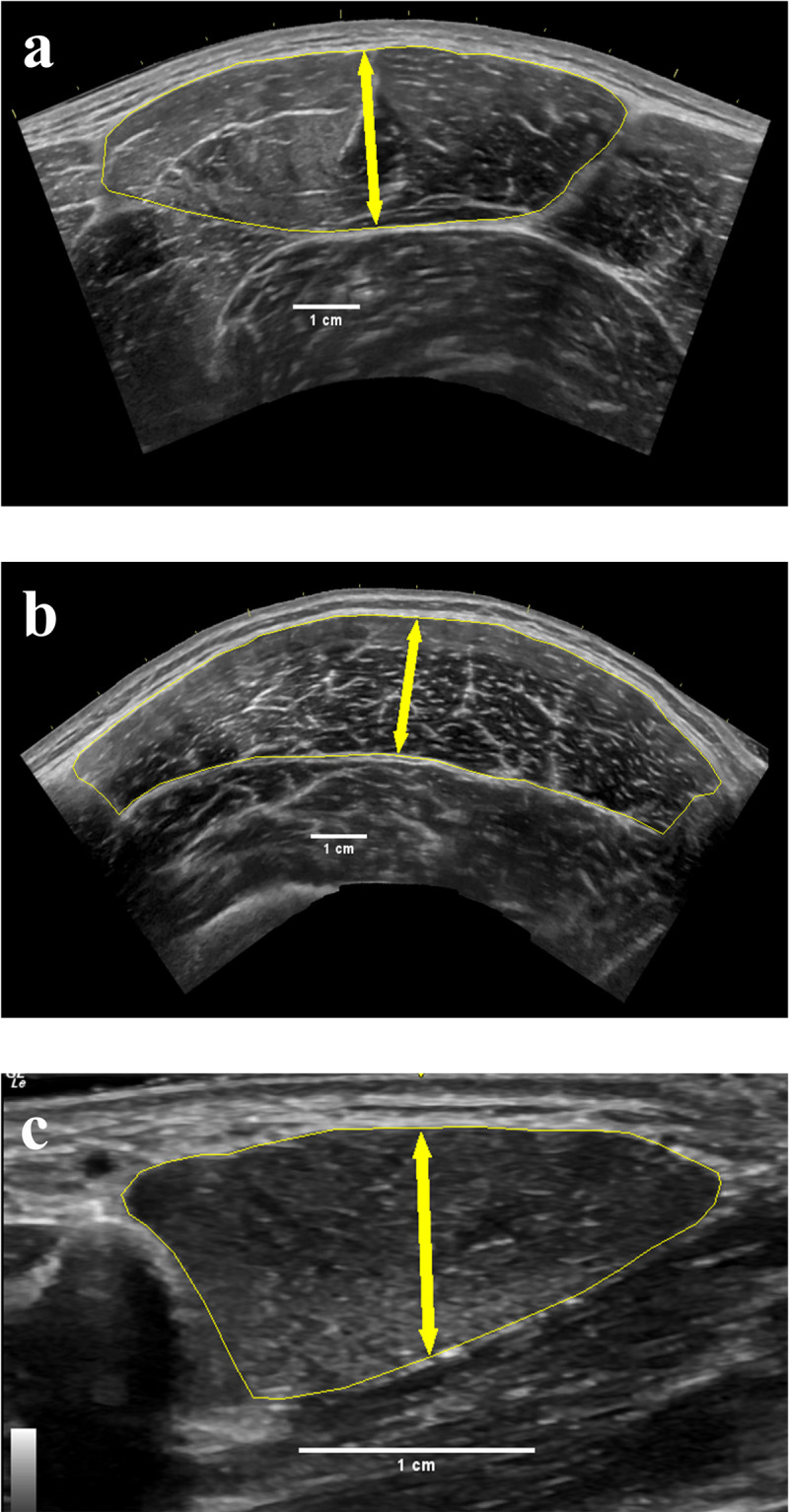
Ultrasound-derived images of the three skeletal muscles utilized in the ImageJ analyses; a = rectus femoris; b = vastus lateralis; c = first dorsal interosseus. The yellow outline represents CSA while the double-sided arrow represents muscle thickness.

Image analyses were conducted utilizing readily available software (ImageJ, National Institutes of Health, Bethesda, MD, USA). Raters were provided with a 2-page PDF document that detailed the procedures for measuring CSA, MT, and EI for each selected muscle. Screenshot examples of each step in ImageJ were embedded within this document along with step-by-step written instructions. All raters were instructed to follow the instructions three separate times per image. Prior to analysis of each image, the image was scaled from pixels to cm with ImageJ’s straight-line function using a known distance of 2 cm. MT was determined as the greatest thickness at the horizontal midpoint of the muscle belly between the superficial and deep aponeuroses, perpendicular to the femur for the VL and RF and perpendicular to the second metacarpal for the FDI, using ImageJ’s straight-line function. The midpoint of the muscle belly was determined from a line drawn from the lateral and medial borders of the muscle using ImageJ’s straight-line function. The straight-line function provides an indicator at 50% of the distance of any drawn lines and MT was taken at this point. Muscle CSA was assessed using ImageJ’s polygon function by manually tracing the border of the muscle belly. EI was determined as the mean gray-scale analysis in ImageJ of the muscle’s whole CSA region of interest (AU) (black = 0, white = 255). Care was taken to ensure that muscle fascia was not included in the MT and CSA analyses. An example of the border outlines for the RF, VL, and FDI are presented in Fig 1 (a, b, and c, respectively). The raters all experienced the same level of training on the software and were somewhat familiar of the functions, however, could be considered novice as their interaction with the software was limited for musculoskeletal ultrasound image analysis.

#### Raters

Three raters completed the image analyses in ImageJ. As stated previously, the images had been collected from previous projects in our laboratory by experienced sonographers. However, the raters would be classified as novices for ultrasound image analysis. Rater 1 had minimal experience with ultrasound in general and had only seen non-panoramic (single transverse plane) images. Rater 2 had some experience with panoramic ultrasound image analysis (<50hrs). While Rater 3 had no previous experience with ultrasound image analysis. Each rater analyzed the images on their own, without any of the other raters present. Raters did not analyze a single image consecutively; they analyzed the entirety of the images before repeating an image for the second and third analyses. In some instances, a rater would only analyze some images at any given time but not the entire set of images in one sitting.

### Statistical analyses

All raters performed the image analysis three times per given image to determine their individual reliability (intra-rater). Averages among the intra-rater values were calculated and subsequently used to calculate inter-rater reliability. These were conducted separately for each muscle; the muscles were not collectively analyzed due to the limited number of images per muscle imaged from the previous studies. One-way ANOVAs were first conducted to examine systematic inter-rater variability in CSA, MT, and EI for each muscle to investigate if there were significant differences between raters (main effect for rater) using the mean values across analysis trials [23]. Separate repeated-measures ANOVAs were then conducted per rater, outcome, and muscle to assess for a main effect for trial (trial 1 v trial 2 v trial 3). In the event of a significant main effect, pairwise comparisons were conducted with Bonferroni corrections. Intra-class correlation coefficients (ICC), the standard error of measurement (SEM), and the minimal difference (MD) needed to be considered real were calculated for each muscle and variable as well [1, 23]. The ICC model “2,*k*” was utilized to calculate the ICCs for both intra- and inter-rater reliability [23, 24]. Model 2,*k* was chosen as it has been suggested that the ICCs generated with this model can be generalized to other laboratories and testers and uses greater than two raters and time points [1]. Inter- and intra-rater reliability was performed for the CSA, MT, and EI of the VL, RF, and FDI. The SEM was calculated from the square-root of the mean square error (MS_E_). The MD needed to be considered real was calculated from the SEM via equations put forth by Weir [23]. The calculations for ICC, SEM, and MD were performed using a custom-written Microsoft Excel spreadsheet (Microsoft Excel, Microsoft, Redmond, WA, USA). The ANOVA was conducted in IBM SPSS v 27.0 (IBM, Armonk, NY, USA). An alpha level of p < 0.05 was considered statistically significant for all analyses.

## Results

### Intra-rater reliability

*R*eliability within each rater (Table 1) was very high and ranged from an ICC_2,*k*_ of 0.961–0.999. The one-way ANOVA indicated a significant main effect for trial for rater 1 VL EI measurements (p = 0.002), as trial 3 was significantly lower than trials 1 (mean difference = 1.06±1.4 AU; p = 0.030) and 2 (1.04±1.3 AU; p = 0.024). For rater 2 there was a main effect for trial in VL MT (p = 0.016) as trial 3 was lower than trial 1 (mean difference = 0.06±0.7 cm; p = 0.036). Also, for rater 2, there was a main effect of trial for CSA of the VL (p = 0.010), however the pairwise comparisons indicated no differences between trials (p = 0.081–0.862). Lastly, rater 3 had a main effect for trial in VL CSA (p = 0.019) as trial 2 was less than trial 1 by a mean difference of 0.19 cm^2^ (p = 0.032) and RF EI (p = 0.016) as trial 1 was greater than trial 2 by 0.26±0.36 AU (p = 0.038). Systematic variability was not present in any of the other outcomes for any of the three raters (p > 0.05).

**Table 1:** Individual rater analysis, rater average measurements, SEM (plus expressed as a percentage of the mean), and MD for all muscle characteristics.

		Vastus Lateralis			Rectus Femoris			First Dorsal Interosseus		
	Analysis	MT (cm)	CSA (cm^2^)	EI (AU)	MT (cm)	CSA (cm^2^)	EI (AU)	MT (cm)	CSA (cm^2^)	EI (AU)
**Rater 1**	*1*	2.29±0.34	26.38±5.76	61.16±17.78^a^	2.22±0.46	10.80±3.76	65.15±21.54	0.88±0.10	1.96±0.41	48.61±4.49
	*2*	2.33±0.35	27.01±6.12	61.18±18.02^a^	2.24±0.46	10.84±3.89	65.20±21.12	0.88±0.09	1.91±0.34	48.57±5.11
	*3*	2.30±0.33	26.61±5.87	62.22±17.93	2.19±0.45	10.78±3.70	65.38±21.74	0.88±0.09	1.90±0.34	48.47±5.11
	***Average***	**2.30±0.33 **	**26.67±5.79 **	**61.52±17.50**	**2.22±0.44**	**10.81±3.70 **	**65.24±20.98**	**0.88±0.09 **	**1.93±0.35 **	**48.55±4.77 **
	***SEM***	0.08 (3.3%)	0.89 (3.3%)	0.83 (1.3%)	0.05 (2.2%)	0.49 (4.5%)	0.56 (0.8%)	0.02 (2.7%)	0.11 (5.8%)	0.80 (1.6%)
	***MD***	0.21	2.46	2.29	0.13	1.35	1.55	0.07	0.31	2.23
**Rater 2**	*1*	2.33±0.33^a^	27.37±5.31	62.22±18.63	2.32±0.45	11.06±3.74	65.10±21.48	0.96±0.14	1.82±0.42	47.68±5.79
	*2*	2.32±0.30	27.50±5.39	62.39±18.45	2.32±0.48	11.03±3.77	65.06±21.58	0.95±0.13	1.82±0.43	47.78±5.74
	*3*	2.27±0.35	26.44±5.78	61.68±17.85	2.28±0.46	10.91±3.68	64.99±21.42	0.95±0.14	1.83±0.42	47.95±5.95
	***Average***	**2.31±0.32 **	**27.11±5.39 **	**62.10±17.89**	**2.31±0.32** ^**c**^	**11.00±3.65 **	**65.05±21.00**	**0.95±0.13 **	**1.82±0.41 **	**47.80±5.66 **
	***SEM***	0.06 (2.3%)	0.97 (3.6%)	1.41 (2.3%)	0.07 (2.8%)	0.13 (1.1%)	0.36 (0.06%)	0.04 (4.1%)	0.02 (1.1%)	0.29 (0.6%)
	***MD***	0.15	2.67	3.92	0.18	0.35	1.01	0.11	0.06	0.81
**Rater 3**	*1*	2.29±0.34	26.20±5.92	60.56±17.86	2.23±0.44	10.84±3.67	64.06±21.63	0.89±0.08	1.94±0.32	48.80±4.87
	*2*	2.30±0.35	26.01±5.85^b^	60.62±17.98	2.23±0.46	10.82±3.70	63.79±21.44^b^	0.88±0.08	1.95±0.35	48.26±4.78
	*3*	2.28±0.34	26.04±5.80	60.55±17.93	2.23±0.45	10.82±3.66	63.97±21.62	0.88±0.08	1.94±0.34	48.17±4.78
	***Average***	**2.29±0.33 **	**26.08±5.73** ^**c**^ ** **	**60.57±17.51** ^**c**^	**2.23±0.44**	**10.83±3.59 **	**63.94±21.07** ^**c**^	**0.88±0.08 **	**1.94±0.32 **	**48.41±4.68 **
	***SEM***	0.04 (1.6%)	0.19 (0.7%)	0.39 (0.6%)	0.03 (1.2%)	0.10 (0.9%)	0.24 (0.4%)	0.03 (3.2%)	0.03 (1.3%)	1.0 (2.1%)
	***MD***	0.10	0.52	1.08	0.07	0.27	0.66	0.08	0.07	2.76

MT = muscle thickness; CSA = cross-sectional area; EI = echo intensity; AU = arbitrary units.

^a^significantly different than trial 3 for that rater

^b^significantly different than trial 1 for that rater.

^c^significant inter-rater difference.

### Inter-rater reliability

Inter-rater reliability statistics measured by ICC_2,*k*_, SEM, and MD for the MT, CSA, and EI of all three muscles are presented in Table 2. Some differences between raters were present in MT of the RF (p < 0.001) and FDI (p = 0.043), CSA of the VL (p = 0.006), and EI of the VL (p < 0.001) and RF (p < 0.001) as the repeated measures ANOVA model indicated significant differences among raters. Rater 2 measured greater MT of the RF than rater 1 by 0.91±1.0 cm (p = 0.013) and rater 3 by 0.78±0.8 cm (p = 0.04). Although there was a main effect for rater on FDI MT (p = 0.043), the pairwise comparisons indicated there were not significant differences between any of the raters (p = 0.24–0.99). The main effect for rater in VL CSA indicated rater 3 measured lower CSA by 0.58±0.8 cm^2^ (p = 0.041) than rater 1 and 1.02±1.2 (p = 0.019) lower CSA compared to rater 2. The main effect for rater of the VL EI measurements indicated rater 3 was significantly lower than rater 1 (mean difference = 0.95±0.7 AU, p < 0.001) and rater 2 (mean difference = 1.53±1.5 AU, p = 0.004). Lastly, for RF EI, rater 3 was significantly lower than rater 1 (mean difference = 1.30±0.8 AU, p < 0.001) and rater 2 (mean difference = 1.11±1.0 AU, p = 0.003). No other differences existed for any of the other outcomes of the selected muscles. Inter-rater reliability was found to be highly consistent in the VL (ICC_2,*k*_ range = 0.993–0.998) and the RF (ICC _2,*k*_ range = 0.984–0.999) across the three raters. For the FDI, MT had an ICC_2,*k*_ of 0.614, while CSA and EI were 0.842 and 0.962, respectively.

**Table 2:** Inter-rater reliability for muscle thickness, cross-sectional area, and echo intensity.

		VL (n = 15)	RF (n = 15)	FDI (n = 12)
**MT**	***p***	0.497	<0.001	0.043
	**ICC**_**2,k**_	0.993	0.984	0.614
	**SEM (cm)**	0.048	0.056	0.075
	**MD (cm)**	0.133	0.156	0.208
**CSA**	***p***	0.006	0.189	0.387
	**ICC**_**2,k**_	0.987	0.997	0.842
	**SEM (cm** ^**2**^ **)**	0.796	0.306	0.223
	**MD (cm** ^**2**^ **)**	2.206	0.848	0.619
**EI**	***p***	<0.001	<0.001	0.547
	**ICC**_**2,k**_	0.997	0.999	0.962
	**SEM (AU)**	0.937	0.694	1.751
	**MD (AU)**	2.596	1.923	4.854

EI = echo intensity; MT = muscle thickness; CSA = cross-sectional area; SEM = standard error of the measurement; MD = minimal difference needed to be considered real; VL = vastus lateralis; RF = rectus femoris; FDI = first dorsal interosseous; AU = arbitrary units.

## Discussion

The novelty of this study included the use of three separate raters analyzing the same previously captured images in triplicate, the assessment of multiple image variables (MT, CSA, and EI), and the inclusion of both lower- and upper-limb skeletal muscles. As research team members of laboratories vary throughout time, this study was designed with the aim of focusing on only the post-image acquisition portion of ultrasound assessment. The primary outcomes of this study included high reliability, measured from the reliability statistics of the ICC and SEM between multiple raters. Despite the high reliability outcomes, some differences among the three raters were identified and referred to as ‘systematic variability’ where a rater was consistently different than the other raters. These significant differences were present in RF MT measurements, muscle CSA of the VL, and EI of the VL and RF. Each of the three raters, independently, had high reliability values for each outcome and muscle. However, there were some differences present between trials for each rater, but this was dependent upon muscle and measurement type, and never exceeded the minimal difference needed to be considered real. For the two quadriceps femoris muscles, the inter-rater ICCs ranged from 0.993–0.999 with SEMs of 1.07–2.50% of the mean (S1 File). While for the FDI, inter-rater ICCs were lower (0.614–0.962) with higher SEMs of 3.63–11.77% of the mean. Overall, these results suggest that when utilizing three raters for the process of ultrasound image analysis, the reliability of the results can depend upon the muscle and measurement investigated.

Rater 3 had the least amount of experience with ultrasound image analysis and reported some measurements that were different from the other raters. However, the SEM for rater 3 was typically lower than the other raters (Table 1). Previous literature has emphasized the effect of experience level on image analysis [15, 25, 26], yet with established, written guidelines supporting the image analysis for any raters, the overall ICCs among and within raters were very high. Takahashi et al. measured MT of the RF with two raters and reported an ICC of 0.70, however tests of systematic error were not reported [26]. Interestingly, Rabello et al. had similar results to the present data when assessing RF EI between two raters with an inter-rater ICC of 0.98 and similarly reported significant differences between the two raters [25]. Similarly, Sobolewski et al. measured CSA and EI of the RF and VL between two raters, reported no significant differences between raters for either muscle or variable, and reported lower ICCs and SEMs than the present study [27]. In fact, the MD for VL CSA in the present study (1.89 cm^2^) was similar to that of Sobolewski et al (1.88 cm^2^), despite differences in total CSA between these two samples [27]. Overall, the MD, which is a measure of the smallest detectable change needed to be considered real, was low for all outcomes and muscles in the present study. It is also worth noting that our analyzed images had greater CSA and higher EI than Sobolewski et al. [27], potentially increasing the amount of error possible. This difference may have contributed to the limited differences observed between raters in the present study.

For intra-rater reliability, ICCs ranged from 0.961 to 0.999 and SEMs of 0.4–4.5% of the mean, which is greater than some previous works [27] and similar to others [7]. However, slight systematic variability was detected in the EI of the VL for rater 1, the CSA and MT of the VL for rater 2, and the VL CSA and RF EI for rater 3 (S1 File). Similar to the differences observed in inter-rater reliability, these significant differences could be due to factors such as the experience level of the rater [15]. Further, as revealed by the pairwise comparisons, raters had somewhat different measurements between trials, particularly in the VL. The VL is a particularly large muscle to fully differentiate from the underlying vastus intermedius near the more posterior aspect and also was the largest muscle examined in the present study. As the borders of the VL may be difficult to determine based on these factors [13], this may explain why trials differed within raters, particularly for CSA and EI. When analyzing muscle architecture such as these, the mean of multiple analyses may be the most accurate estimation of CSA and EI, supporting our previous notion that more than 3 trials of analyses are needed to reduce the error observed and wash-out any erroneous measurements across trials. Nevertheless, the SEM and MD for intra-rater values were small, similar to the inter-rater results. Furthermore, these all resulted in reliable analyses determined by the ICC and SEM, in agreement with previous literature [12, 16, 27].

To the authors’ knowledge, this is the first study to assess reliability of the panoramic ultrasound image analysis measurements of the FDI. Overall, the ICC_2,*k*_ of the FDI was 0.614 for MT, 0.842 for CSA, and 0.962 for EI, which are noticeably lower than the two lower-body muscles assessed. Yet, there were no significant differences between or within the raters for FDI image analyses. The lower reliability statistics could be due to the FDI being in an uneven body region on the hand [10] that presents challenges to analysis using obvious anatomical landmarks, unlike the femur, for example, when measuring the quadriceps muscles. A previous study utilized brightness-mode ultrasound and the ultrasound device’s built-in software to analyze the images to examine the reliability of CSA measurements of the FDI on two separate occasions which resulted in an ICC of 0.979 [28]. With these methodological differences and the resulting ICCs determined from the analyses of these two studies, it is difficult to directly compare results. Therefore, it is imperative to continue attempt to standardize ultrasound image acquisition and analysis for different skeletal muscles to easily compare results across laboratory groups, populations, and studies.

Despite the high reliability noted from the ICCs, SEMs, and MD, there were still some differences between and within the raters for some, but not all, of the muscles and variables. A complete data set are available in supporting information (S2 File). It should be emphasized and noted that reliability statistics (ICC and SEM) are not the same as an ANOVA and interpretation will differ based on test, use, and calculations [23, 24, 29]. For example, the ICC represents relative consistency while the SEM is a measure of absolute consistency [23]. With this in mind, it is plausible to have statistically significant differences and very high ICCs. For example, Rabello et al. [25] identified significant differences in RF EI between two raters but the ICCs ranged from 0.98–0.99. Further, Weir suggested for an ANOVA to detect significant differences, the mean differences between trials (or raters in the present study) must be quite large, while the error term (which represents the interaction between raters and ratings) is small, or both [23]. Therefore, if the goal is to compare ultrasound image analysis results across multiple raters, it may be warranted to adjust the number of measurements performed by each rater. Future studies and lab groups may benefit from having raters repeating measures until a plateau is observed [23] or a training period in which raters are provided “practice” or control images before performing study-related data analysis, similar to including a familiarization trial in experimental studies. This would help establish a baseline for the specific rater.

The SEM values in the present study ranged from 0.05–0.07 cm for MT, 0.23–0.80 cm^2^ for CSA, and 0.60–0.94 AU for EI of the quadriceps muscles measured, and these were all relatively small expressed relative to the mean (range: 0.4–5.8%). Regarding the results of the SEM and to provide the reader with appropriate discourse in interpretation and comparison to other studies, it is worthwhile to note there are multiple ways to assess the SEM (S1 File). The present study equated SEM as the MS_E_ from the ANOVA results, as suggested by Weir [23] and Hopkins [30]. Rabello et al. [25] calculated the intra-rater SEM as the SD multiplied by the square root of 1 –the ICC for RF EI and had SEMs of 3.97 AU and 6.49% of the mean, which are greater than the present study’s results. While Takahashi et al. [26] and Rosenberg et al. [12] used the same equation as the present study for VL and RF CSA and EI. Using the present study’s data as an example, our SEM value for inter-rater reliability (calculated from the square root of the MS_E_) for the CSA of the VL was 0.796 cm (~2.9% of the grand mean), but if calculated as the square root of 1 –ICC_2,*k*_ the SEM would then be 1.144 cm (~4.2%). Although these differences may not occur in each situation or might not severely impact the interpretation of the SEM, dependent upon the variable assessed, it is still pertinent to acknowledge that there are deviations in how reliability statistics can be calculated.

Limitations of the present study include the lack of descriptive characteristics of the participants. The use of different computers with potentially differing screen resolution, size, etc., and differing cursor mobility (i.e., trackpad v. physical mouse), and many other computer-related differences could have resulted in any differences between raters within this study. Future studies may benefit from standardizing these variables, however, we felt it made the present study the most realistic for academic research settings as the post-acquisition image processing is often the limiting step in research laboratories with research assistant (student or post-doctoral) turnover. Despite these limitations, the reported ICCs within each rater across three separate analyses suggest that consistent instructions and guidance on ultrasound image analysis allowed each rater to reliably analyze the selected outcomes.

## Conclusion

In conclusion, the results of the present study suggest that multiple raters can reliably estimate MT, CSA, and EI of the VL, RF, and FDI with standardized post-acquisition processing methods. The use of three separate raters with limited experience was unique and novel in this present study. Although systematic variability was present in some of the selected muscles and outcomes, the overall reliability was still very high. The FDI had the lowest reliability of the three muscles, and this was most likely due to its unique location on the hand, yet it remained at an acceptable level of reliability. Uniquely, this study was the first to assess panoramic ultrasound reliability of the FDI and can provide further insight into ultrasound analysis of the skeletal muscles when the muscle cannot be captured within one single longitudinal image. Future studies should continue to investigate the impact of raters with varying levels of experience, various skeletal muscles, and independently per lab group on the reliability of US quantification of skeletal muscle characteristics.

## Supporting information

S1 File Standard error of the measurements table for inter-rater reliability presented through two equations and figure of ultrasound related measurements per trial, muscle, measurement, and rater. Black lines represent the mean values while grey lines represent each image.
(PDF)

S2 File Supporting data Excel file. All data by the three raters used in analyses.
(XLSX)
