# Supplementary material for: Skeletal muscle analysis of panoramic ultrasound is reliable across multiple raters
Source: PLoS One. 2022 May 2;17(5):e0267641. doi: 10.1371/journal.pone.0267641 (PMC9060372; doi:10.1371/journal.pone.0267641)

S1 Table. Standard error of the measurements for inter-rater reliability presented through two equations.

		**VL**	**RF**	**FDI**
**MT**	**Grand mean (of 3 raters)**	2.299	2.253	0.906
	**SEM (sqrt of MSE) (cm)**	0.0467	0.0563	0.075
	**% of mean**	2.08%	2.50%	8.28%
	**SEM (SD * sqrt (1 – ICC)) (cm)**	0.0478	0.0993	0.1169
	**% of mean**	2.03%	4.41%	12.91%
**CSA**	**Grand mean (of 3 raters)**	26.619	10.878	1.8956
	**SEM (sqrt of MSE) (cm^2)**	0.7959	0.3063	0.2232
	**% of mean**	2.99%	2.82%	11.77%
	**SEM (SD * sqrt (1 – ICC)) (cm^2)**	1.144	0.3296	0.2585
	**% of mean**	4.30%	3.03%	13.64%
**EI**	**Grand mean (of 3 raters)**	61.398	64.745	48.255
	**SEM (sqrt of MSE) (AU)**	0.9365	0.6937	1.7512
	**% of mean**	1.53%	1.07%	3.63%
	**SEM (SD * sqrt (1 – ICC)) (AU)**	1.5781	1.3672	1.7408
	**% of mean**	2.57%	2.11%	3.61%

S1 Fig. Muscle thickness measurements per rater

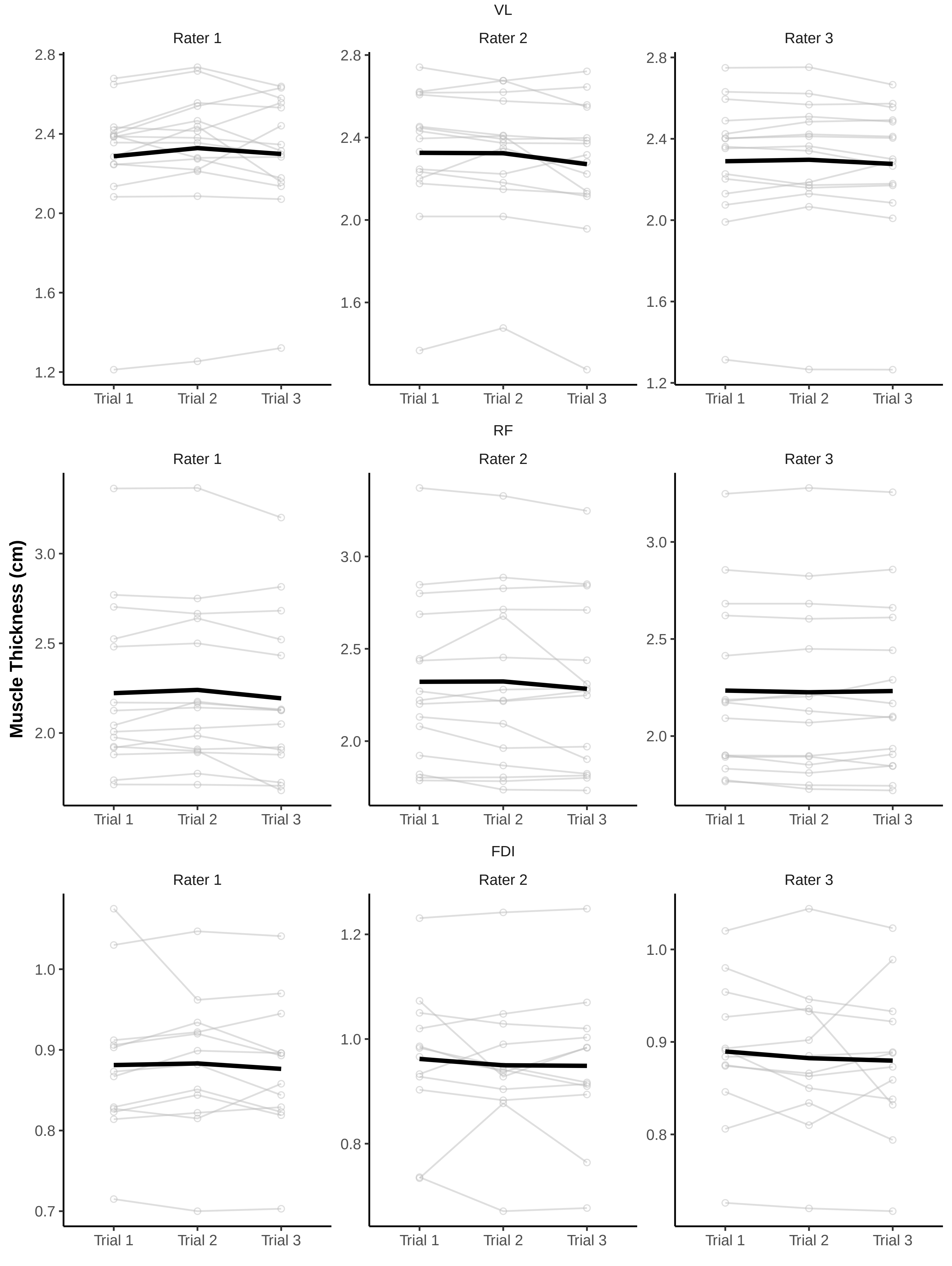

S1 Fig. Muscle cross−sectional area measurements per rater

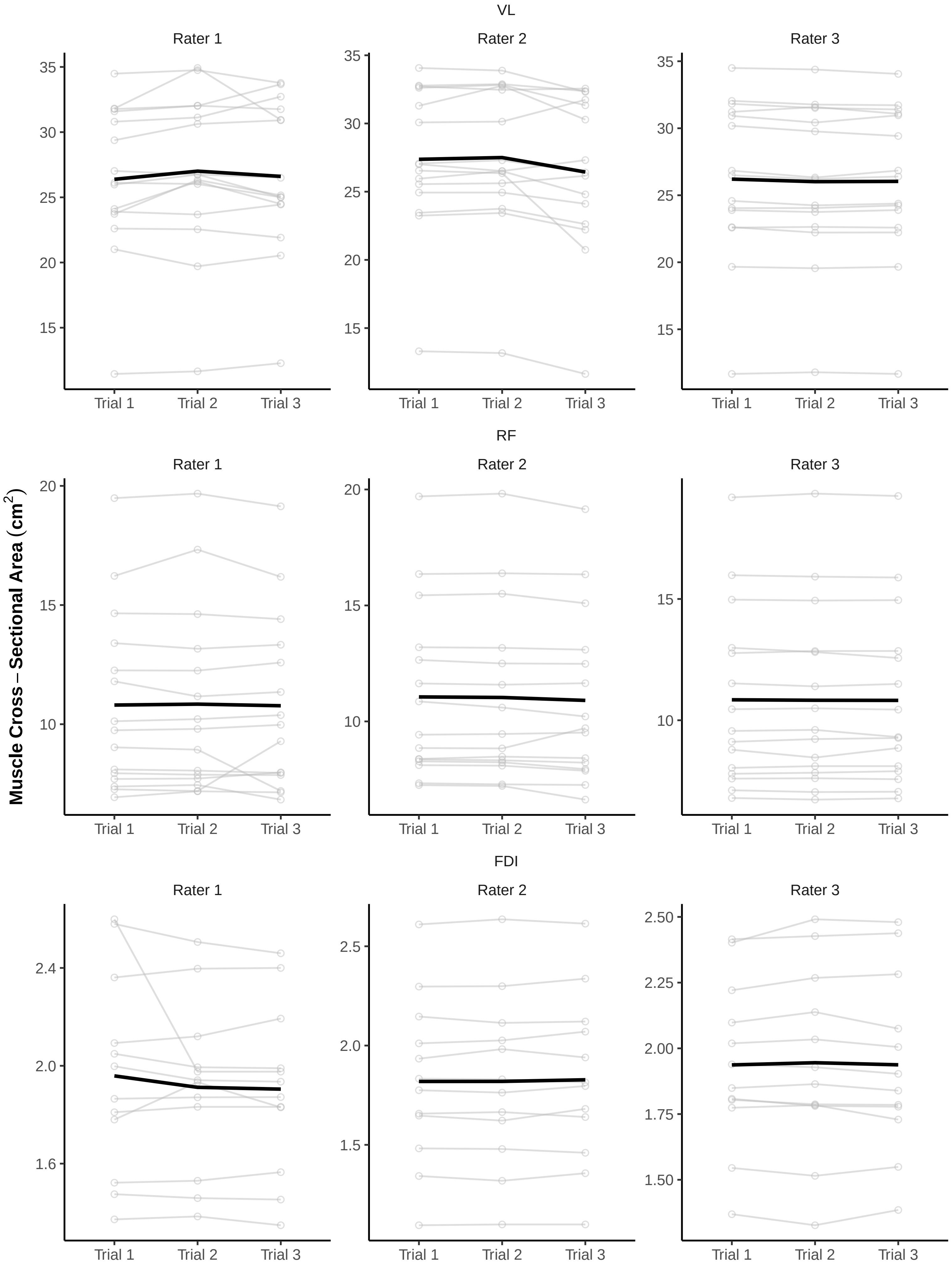

S1 Fig. Echo intensity measurements per rater

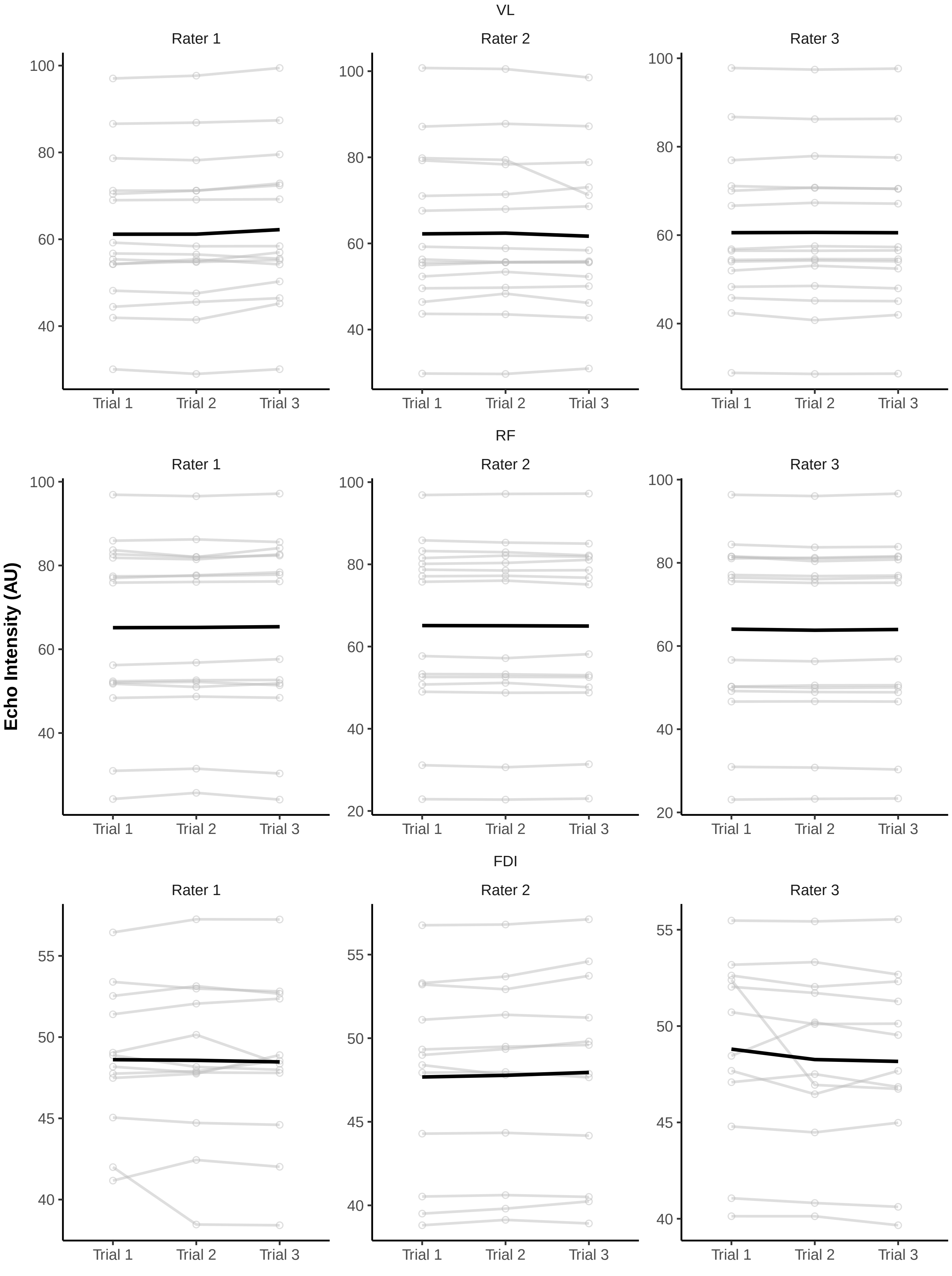

Supplement: S1 File — Black lines represent the mean values while grey lines represent each image. (PDF) [file pone.0267641.s001.pdf]
